# Knowledge and Attitudes towards Palliative Care: Validation of the Spanish Version of Questionnaire on Palliative Care for Advanced Dementia

**DOI:** 10.3390/healthcare10040656

**Published:** 2022-03-31

**Authors:** Elena Chover-Sierra, Pilar Pérez-Ros, Iván Julián-Rochina, Carol O. Long, Omar Cauli

**Affiliations:** 1 Nursing Department, University of Valencia, 46010 Valencia, Spain; elena.chover@uv.es (E.C.-S.); maria.p.perez-ros@uv.es (P.P.-R.); ivan.julian@uv.es (I.J.-R.); 2 Nursing Care and Education Research Group (GRIECE), University of Valencia, 46010 Valencia, Spain; 3 Internal Medicine, Consorcio Hospital General Universitario de Valencia, 46014 Valencia, Spain; 4 Frailty Research Organized Group (FROG), University of Valencia, 46010 Valencia, Spain; 5 “Palliative Care Essentials” Research Institute, Fredericksburg, VA 22407, USA; carollongphd@gmail.com

**Keywords:** Alzheimer’s disease, neurodegenerative diseases, pain, end-of-life care, nursing

## Abstract

Background: Palliative care is essential in the care of people with advanced dementia, due to the increasing number of patients requiring care in the final stages of life. Nurses need to acquire specific knowledge and skills to provide quality palliative care. The Questionnaire on Palliative Care for Advanced Dementia (qPAD) is useful for assessing knowledge and attitudes toward palliative care, but its adaptation to the Spanish language and analysis of its effectiveness and usefulness for the Spanish culture is lacking. Objective: To report on the Spanish language adaptation and psychometric analysis of the qPAD. Methods: The Questionnaire on Palliative Care for Advanced Dementia Spanish version (qPAD-SV) was obtained from a process that included translation, back-translation, comparison with other language versions, expert review, and pilot study. Content validity, criterion validity, and reliability of the questionnaire were analyzed. The difficulty and discrimination indices of items composing the knowledge subscale were also calculated. Results: Adequate content validity index obtained after the analysis of qPAD-SV by a heterogeneous group of experts was found (overall CVI = 0.96; 0.95 for the Knowledge subscale and 0.99 for the Attitudes subscale). Significant correlations with the Palliative Care Knowledge test (rho = 0.368, *p* < 0.001) and Self-Efficacy in Palliative Care Scale (rho = 0.621, *p* < 0.001) show an adequate criterion validity. Cronbach’s alpha coefficients for the Knowledge subscale (0.60) and the Attitudes subscale (0.91) supported the reliability of the qPAD-SV. The questionnaire had an overall difficulty index of 0.71, with three items that could be considered difficult or very difficult, and eleven items that could be considered very easy. Discussion: Although it shows internal consistency, validity, and difficulty indices similar to those obtained by qPAD versions in other languages, a reformulation of the items with lower content validity or discrimination indices and those that show difficulties in their comprehension is an aspect to be taken into account to improve this tool. Conclusions: The qPAD-SV is a useful instrument in Spanish to measure the knowledge of Spanish nurses in palliative care and is suitable for international comparisons.

## 1. Introduction

More than 55 million people live with dementia worldwide. Alzheimer’s disease is the most common form of neurocognitive disorder (widely known as dementia) (60–70% of cases). It is the major cause of disability and dependency among older people globally and has physical, psychological, social, and economic impacts, not only for people living with dementia but also on their careers, families, and society at large. It is not part of the aging process, although it mainly affects older people. The WHO recognizes dementia as a public health priority [1].

Most people with dementia live at home with an informal caregiver and in more advanced stages, in the absence of perceived adequate treatment and care at home by the families, they are transferred to residential care homes to address increasing care deficits [2]. Roughly 65% of people in residential care homes have dementia, most of them being people over 80 years of age [3]. 

Knowing that dementias are a group of chronic conditions for which there is no cure, it must be assumed that palliative care that focuses on the relief of the symptoms and physical and mental stress of living with a serious illness, is necessary for advanced dementia [4]. A guide about palliative care decisions for nursing home staff based on a project on the end-of-life decisions for people with dementia has been proposed [5,6]. 

Therefore, people in the advanced stages of dementia have a high demand for care, mainly palliative care, but despite the availability of generic definitions of palliative care, there is a lack of training because professionals still state that it is unclear what palliative care people with advanced dementia need and when [7]. In addition, there is an under-referral of people with advanced dementia compared to people with other pathologies that utilize palliative care such as cancer or heart disease [8].

In a white paper on palliative care in advanced dementia, the European Association of Palliative Care outlined the key pillars of palliative care and identified the need for education of the health care team, and recommended dementia care practices and skills that augment palliative care that are defined for this population [9].

There is a lack of knowledge of advanced dementia care worldwide both in the general population and among health professionals [10,11]. Dementia education would allow the implementation of preventive measures at early stages, strategies that allow active attention to care deficits once the disease is established and home care strategies for family members [12]. In undergraduate health education courses, there are generic modules on mental health or geriatrics but no subject matter in dementia and similarly there are few postgraduate courses in dementia [13]. The under-referral of people with advanced dementia to palliative care among physicians is related to a deficit in palliative care knowledge and the lack of inclusion of families in decision-making [14]. Nurses working with people with advanced dementia in long-term care (LTC) also express the need for training because they have little knowledge about dementia [15,16,17], and they do perceive that these people require more hands-on care. With increasing demands in the workplace and time constraints in the delivery of essential nursing care, it becomes a challenge to meet the needs of people with advanced dementia and at the end of life [7]. Dementia care training is necessary for all professionals working in hospital services, residential care, and primary care and it is wrongly felt that training in dementia care should only be received by professionals working in nursing homes [18]. This is because most of the people who use hospital services are older people and home care in primary care is mainly for older people, in LTC most of the residents live with dementia [19]. 

Lack of knowledge in the management and implementation of palliative care for people with advanced dementia in professional staff decreases the quality of care, worsens the disease progression, the prognosis of dementia, and decreases professionals’ positive attitudes towards palliative care [8]. It also increases unnecessary and/or inappropriate interventions. People with advanced dementia often have increased recurrent hospitalizations, antimicrobial treatment, such as the use of antibiotics for unnecessary pneumonia treatment; increased nutritional problems due to a lack of knowledge of dysphagia management that often include the use of methods to augment nutrition and hydration through the inappropriate use of feeding tubes, largely due to the lack of training and understanding by health care professionals [7]. In contrast, early referral to palliative care services is associated with increased use of inpatient palliative care services, prevention of overly aggressive treatments, and improved comfort and quality of life for people with dementia and their families [20].

Whether there is evidence on which symptomatology is mainly associated with the need for the initiation of palliative care in people with advanced dementia needs to be established [6,21]. It is necessary to know what knowledge and attitudes professionals so to propose strategies for improvement in dementia care practices [4,22,23].

Several tools are available to quantify knowledge, skills, and attitudes about palliative care in health professionals [24] and there are also instruments to assess knowledge about dementia [25]. However, there is only one instrument that is able to measure knowledge and attitudes in palliative care for advanced dementia and that is the qPAD [26].

Long et al. [26] developed the qPAD based on the measurement of knowledge through several items of the Knowledge of Alzheimer’s Test (KAT) and the assessment of attitudes through the End-of-Life Care Decision Questionnaire II (EOLCDII) [26,27]. In the qPAD, each item is scored as “Agree or Disagree or I Don’t know” in the part of knowledge about palliative care and a 5-point Likert test, with 1 indicating strongly disagree and 5 strongly agree in the part of attitudes towards palliative care. The qPAD comprises the measurement of knowledge on three factors: (1) Anticipating Needs (coefficient α = 0.75), (2) Preventing Negative Outcomes (coefficient α = 0.73), and (3) Insight and Intuition (coefficient α = 0.58). Three factors are assessed for the measurement of attitudes: (1) Job Satisfaction (coefficient α = 0.90), (2) Perceptions and Beliefs (coefficient α = 0.64), and (3) Work Setting Support of Families (coefficient α = 0.67) [26].

The qPAD has been used in a number of studies around the world, from the analysis of knowledge and attitudes in Taiwan [8], Japan [28], and Australia [29,30], following the implementation of an intervention in nursing home staff [30,31,32] and previous validation of the questionnaire in the Japanese language [33]. In addition, two studies used the qPAD as part of the construct validation of their own instrument [24,34].

In Spain, the prevalence of dementia is progressively increasing in age bands between 1.07% among 65–69 year olds; 3.4% among 70–74 year olds; 6.9% among 75–79 year olds; 12.1% among 80–84 year olds; 20.1% among 85–89 year olds; and 39.2% among those over 90 years of age. The percentage of the population over 65 years of age currently stands at 19.2% of the total and is expected to be 25.2% in 2033. Similarly, 61.5% of people with dementia in nursing homes in Spain have advanced dementia [35]. 

Due to the increasing older population, the prevalence of dementia, and in particular advanced dementia, the number of professionals who are caring for people with advanced dementia, and the lack of knowledge and positive attitudes towards palliative care in advanced dementia revealed in other studies requires further research. The aims of our study were to:

– adapt the qPAD scale in the Spanish language;
– analyze its content and criterial validity;
– analyze the level of difficulty of each item in a pilot study in healthcare professionals.

## 2. Materials and Methods

### 2.1. Elaboration of the qPAD Questionnaire in Spanish and Back Translation of the Spanish Version

The original English version of the qPAD questionnaire was presented to one professional translator and two native Spanish-speaking nurses with knowledge of English who translated the original version of the qPAD questionnaire into Spanish, thus generating three versions of the translation. These translations were performed independently so that no participant was aware of the work of the others. They were asked to make a non-literal translation of the questionnaire, maintaining its original content and purpose. From the three previously prepared translations, a single document was prepared (consensus translation), which was subsequently subjected to a back-translation process, so that it was translated back into English again by two professional translators and a nurse with knowledge of both languages. 

The back-translation was also carried out blindly, so that none of the three knew the translations of the others, although at the end of the process the three were asked to produce a single translation by consensus. After the back-translation phase, we obtained two English versions of the questionnaire, the original version, and the back-translated version, which were compared item by item by the English translator and by the principal investigator of the original version of the qPAD in the English language (Dr. Carol O. Long [26]. At the end of the procedure, a single back-translated version was obtained.

Any difference found between the two English versions was reformulated in the Spanish version; otherwise, the wording of the item was maintained for the final questionnaire. Finally, the Spanish version of the qPAD was obtained and sent to a group of experts for their evaluation. The experts also gave their recommendations to reformulate and clarify any item. Finally, the qPAD-Spanish version (qPAD-SV) was created and tested in a pilot study (Supplementary S1).

### 2.2. Analysis of the Spanish Version of Questionnaire by the Group of Experts

A group of experts consisting of 13 health professionals (registered nurses *n* = 9 and medical doctors *n* = 4) with at least five years of experience in the field of palliative care who care for people with advanced dementia (e.g., members of the Spanish Society of Palliative Care (*n* = 4), working in long-term nursing homes (Geroresidencias La Saleta, Valencia, Spain) (*n* = 6), or in hospital palliative care settings (*n* = 3)) with people with advanced dementia, selected from both the health care and teaching fields, reviewed the Spanish version of the questionnaire. Each of them evaluated the questionnaire individually and sent their evaluation to the researchers. The experts were contacted by means of an e-mail explaining the reasons for the study and requesting their participation. The experts were asked to evaluate each of the items for their appropriateness to the subject of the study, as well as their clarity and comprehension, and to make any contributions they considered necessary for a better understanding of the questionnaire by the professionals who would later have to answer it. These modifications would have been taken into account in the final Spanish version of the qPAD. In addition, they evaluated each of the items on a scale of 1 to 4 (1 = not relevant, 4 = very relevant); these evaluations were subsequently used to calculate the content validity indexes for each item and for the questionnaire as a whole.

### 2.3. Content, Criterial Validity and Difficulty Analysis of the Spanish Version of qPAD

For the content validity analysis, we followed the methodology described by Polit and Beck [36] and used by other authors [37,38] which consists of calculating the content validity of each of the items individually, based on the evaluations made by a group of experts (composed of a minimum of five experts: although the recommendation is to include around ten experts, to avoid the effects of chance as much as possible).

The content validity index (CVI), which is determined for each of the items that make up the questionnaire (I-CVI) and for the overall questionnaire (S-CVI), the S-CVI being the arithmetic mean of the I-CVI, is calculated using the following formula:

CVI = no. of experts who have evaluated the item with 3 or 4 (A)/Total experts (N)

CVI scores equal to or higher than 0.78 are considered acceptable, and values equal to or higher than 0.90 are considered indicative of high content validity [36,37].

The experts’ assessments were also used for the calculation of the modified kappa coefficient (k), which measures the degree of agreement observed between observers, eliminating agreement obtained by chance; thus, it is also an indicator of content validity. This coefficient takes values between 0 and 1. The criteria for assessing agreement determined by the kappa coefficient established were considered (excellent ≥ 0.74; good 0.60–0.74; poor 0.59–0.40). 

The following formula was used to calculate the kappa coefficient (k):

k = (I-CVI) − Pc/1 − Pc

where I-CVI is the internal validity coefficient previously calculated for each of the items and Pc (probability of chance agreement) is the probability that the agreement between observers is due to chance and is calculated by the formula:

Pc = (N!/A!(N − A)) × 0.5^N^

where N corresponds to the total number of experts and A to the number of experts who have evaluated each item as 3 or 4. It is desirable that Pc receives values as small as possible.

Both the CVI and the different Pc and kappa coefficients were calculated using a spreadsheet developed with Excel, in which a database was created with the ratings of the group of experts. The criteria to determine the level of agreement among the experts with the kappa coefficient were calculated (kappa value ≥ 0.74 excellent agreement; 0.60 ≥ kappa value < 0.74 good agreement; kappa value < 0.59 poor agreement) [36].

To assess the criterion validity of qPAD, the score obtained in the part of the Knowledge portion of the qPAD was correlated with the scores obtained in the Self-efficay in PC scale [39], a questionnaire that evaluates the basic knowledge about palliative care, also adapted into the Spanish language [40]. The score obtained in the part of Attitudes portion of the qPAD was correlated with the scores obtained in the Palliative Care Knowledge Test (PCKT) is a questionnaire that evaluates the Self-Efficacy in Palliative Care Scale (SEPC) [41], also adapted by nursing professionals in the Spanish language [42]. These two instruments were used as an external criterion, taking into account the content equivalence with each part of qPAD (Knowledge and Attitudes).

Based on the responses of the group of professionals who participated in the pilot study, an analysis of the reliability of the questionnaire was also carried out by calculating Cronbach’s alpha coefficient. A Cronbach’s alpha value of 0.7 is set as the minimum acceptable value, and lower values indicate a low internal consistency of an instrument, although some authors consider that in the early stages of an investigation and in exploratory research a value between 0.5 and 0.6 may be considered as suitable [43]. 

Finally, and following the postulates of Item Response Theory, the indices of difficulty and discrimination of each of the items that compose the knowledge questionnaire (qPAD part 1) were calculated from the responses of the participants. To measure the level of knowledge, the test items were scored as 1 (correct) or 0 (incorrect/don’t know). Therefore, item difficulty is represented by mean values, low values representing difficult items, and high values representing easy items. Indices of discrimination of the items that compose the questionnaire were calculated using the number of correct answers in the third of the subjects with the best results and in the third with the worst results. Discrimination indices were again calculated with the Microsoft Excel 2013 spreadsheet.

## 3. Results

### 3.1. Review of Experts and Content Validity

After the review of the questionnaire by the group of experts, recommendations emerged for the reformulation of some of the items. These suggestions were discussed with the qPAD author (Dr. Long, co-author of the study and originator of the qPAD English version). Next, we made slight changes to maintain the original meanings of the English version of the qPAD items. For Item 6, the “hand-on care” was translated as an expression similar to “care actions with physical contact”; for Item 7, the Spanish translation of the concept of “take shower” (in the original version in English) may suggest that people with advanced dementia take a shower by themselves, and that could lead to confusion, so a more accurate translation was proposed. similar to “getting a bath” by professionals. For Item 18, the wording conflict arose with the idea of “anticipation of needs”, and what exactly the questionnaire meant by “a daily schedule”. In addition, the idea also arose that not all people with AD live in nursing homes. The concept “anticipation of need” was translated as something similar to “provision of care needs”, and also additional wording was added to clarify that a “daily schedule” was intended to refer to an identical plan of activities for all people with advanced dementia.

The CVI and Kappa values for each of the items that make up the questionnaire, calculated from the experts’ evaluations, are shown in Table 1 and Table 2.

Analyzing the CVI of each item, we see that all of them reach the value of 0.78, which is considered acceptable (in fact, I-CVI range from 0.85 to 1). If we calculate the overall CVI of the questionnaire (S-CVI), we see that it has a value of 0.96, higher than the value defined as acceptable.

As for the kappa index, we can also see that all items have an excellent level of agreement.

Thus, we could say that the qPAD in the Spanish version, or qPAD-SV, presents adequate content validity.

### 3.2. Criterion Validity and Reliability

**Figure 1:**
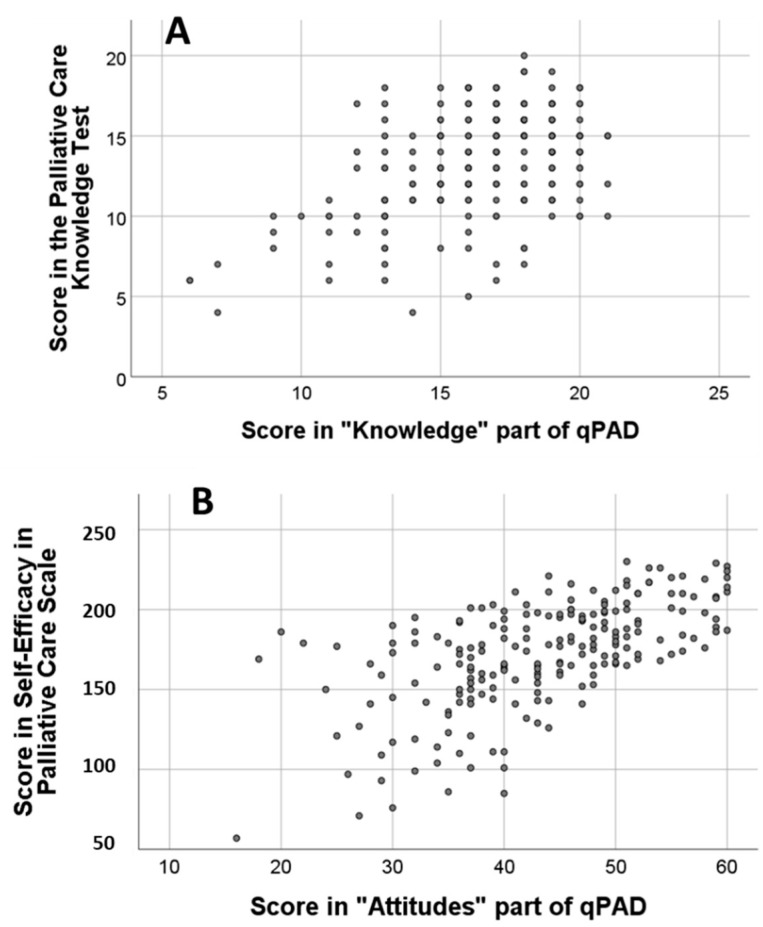
(**A**) Correlation analysis between the score in Palliative Care Knowledge test and the score in the “Knowledge” portion of the qPAD; (**B**) Correlation analysis between the score in Self-efficacy in palliative care scale Palliative Care Knowledge test and the score in the “Attitudes” portion of the qPAD.

Correlations with the instrument (Palliative Care Knowledge Test) used to analyze the criterion validity of qPAD-SV: were good. The scores obtained in the part of Knowledge of the qPAD were significantly correlated with the scores obtained in the Palliative Care Knowledge Test (*p* < 0.001, rho = 0.368) (Figure 1A). The scores obtained from the Attitude items of the qPAD were significantly correlated with the scores obtained in SEPC (*p* < 0.001, rho = 0.621) (Figure 1B).

We calculated the Cronbach index for each subscale of qPAD-SV. The Attitudes subscale attained a Cronbach index of 0.91 (Job Satisfaction 0.91, Perceptions and Beliefs 0.75, and Work Setting Support of Families 0.77), while the Knowledge subscale had a Cronbach index of 0.60.

When analyzing the correlations between the items, it is observed that the Cronbach’s index of Knowledge subscale would increase to 0.62 if we eliminated items 17 or 22, and to 0.64 if we eliminated Item 3. However, we did not consider it necessary to eliminate these items since increasing the reliability index was not that high and maintaining the questionnaire in the same form as the original one would be better to establish comparisons. Besides, these three items had a percentage of wrong answers higher than 40% (item 3) and even higher than 60% (items 17 and 22), so they had been useful to identify these areas of knowledge deficit.

### 3.3. Results of the Pilot Study

A sample of 206 registered nurses (*n* = 154) and physicians (*n* = 52) completed the qPAD Spanish version. Forty-one males (19.9%) and 165 female (80.1%) health professionals participated in the study. The mean age of the participants was 45.1 ± 10.5 years (range 21–66). Moreover, 68.9% reported working with people with advanced dementia, 81.1% reported having training in palliative care, 20% had received this training only during their university education and had not received any additional training.

The percentage of correct and incorrect answers obtained by each participant in the qPAD knowledge questionnaire was calculated; they obtained an average of 70.87% of correct answers (70.87 ± 12.84; range 26.09–91.30) and 29.13% of incorrect answers (29.13 ± 12.84; range 8.70–73.91). This would mean an average of 16.3 points out of a maximum of 23 in the overall score of the questionnaire. 

The percentage of correct and incorrect responses was also calculated for each of the items that make up the questionnaire, resulting in a high variability among them. The items with the highest percentage of correct answers (more than 85%) were items 2, 4, 5, 6, 9, 14, 15, 16, and 20, and those with the highest percentage of errors were items 1, 3, 7, 8, 13, 17, 18, 21, 22, and 23 (more than 30% in the case of items 1, 3, 7, 8, and 23, and more than 50% in the case of items 13, 17, 18, 21, and 22). The percentages of correct and wrong answers and the difficulty and discrimination index for each item composing the qPAD Knowledge subscale, calculated from the number of correct and wrong answers, are shown in Table 1.

Differences in results in the qPAD-SV knowledge subscale were found among participants according to the variable “working with people with advanced dementia” (*p* < 0.001). 

Regarding the qPAD-SV Attitudes subscale, the average score was 43.35 (43.35 ± 9.28; range 16–60) and each subscale scored as follows: Job Satisfaction (24.59 ± 6.62; range 7–35), Perceptions and Beliefs (11.75 ± 2.34; range 5–15), and Work Setting Support of Families (7.01 ± 1.85; range 2–10). The mean score for each item composing this subscale is shown in Table 2. Differences in results in qPAD-SV attitudes were also found among participants according to the variable “working with people with advanced dementia” (*p* < 0.001). 

The analysis of the difficulty index shows that items 2, 4, 5, 6, 7, 8, 9, 10, 11, 12, 14, 15, 16, 19, and 20 can be considered as easy (difficulty index from 0.61 to 0.80) or very easy (more than 0.80), while items 17, 21, and 22 are difficult (difficulty index from 0.21 to 0.40). The average difficulty of the knowledge subscale of the qPAD-SV is 0.71.

The calculation of the discrimination indices for the items included in the Knowledge part of the qPAD showed the best and worst items. Some of the items appear very good (indices above 0.40): items 1,7,13 and 23; good (indices between 0.3 and 0.39): items 10, 11, 18, 19, and 21; fair (indices between 0.2 and 0.29): items 6, 12, 16, 17, 20, and 22; the worst (indices below 0.2): the remaining items of the questionnaire.

## 4. Discussion

The transcultural validation into the Spanish language of the qPAD demonstrated good psychometric properties. Only two articles analyze the psychometric properties of the qPAD validated in other languages as in the Chinese [8] and Japanese versions [33] and the Chinese adaptation showed a CVI of 0.9 for the Knowledge part and CVI of 0.92 for the Attitudes part of the questionnaire. The internal consistency of the Attitudes scale of the Chinese version showed a Cronbach’s α value of 0.82 [8] while the Japanese version was 0.84 [33]. However, while attitudes toward palliative care were as good as in the original English version of the qPAD [26], some of the items on the Knowledge part yielded mixed results in terms of the percentage of correct answers. We observed in our context variable levels of knowledge depending on the specific item. In particular, the items related to weight loss prevention, physical restraint, pain management, anticipation of need, fatigue, and confusion in people with advanced dementia (items 1, 3, 13, 17, 18, 21, 22 of the Knowledge part of the questionnaire) warrant particular effort to improve the knowledge of health care professionals in order to render quality care to people with advanced dementia. We summarize below the main issues related to the items that showed to be “difficult” for the healthcare professionals. 

Regarding nutrition issues in individuals with advanced dementia, one in every two or three people affected will experience severe weight loss, and it may continue even though the common reasons for weight loss have been managed [44,45]. The weight loss can be inevitable due to an advanced disease called cachexia, a normal process caused by advanced diseases such as dementia, cancer, heart, liver, kidney, and lung failure. When people have cachexia, they cannot absorb the nutrients from food, even when they are eating and drinking enough, and the use of special medical diets is no longer required [46]. They lose weight, have no appetite, and become tired and weak. There is some evidence available from retrospective and prospective observational trials on tube feeding and mortality in patients with severe dementia [47,48]. In controlled studies, there is no significant difference between the mortality rates of patients with advanced dementia receiving tube feedings and those without. A Cochrane review, summarizing all relevant previous studies, identified six controlled trials assessing mortality and found no evidence of increased survival in patients with advanced dementia receiving enteral tube feeding [49]. The use of enteral nutrition therapy (ENT) or artificial nutrition and hydration (ANH) is only indicated when there is a risk of malnutrition and severe impairment of the swallowing process, with the possible consequence of aspiration pneumonia [50]. According to ASPEN—American Society for Parenteral and Enteral Nutrition [51], the use of ENT in patients with advanced dementia is classified as not recommendable. 

Regarding the use of physical restraints while in the past it was widespread and fulfilled the criterion for preserving the safety of dependent persons [52,53,54,55] but now it is known that this practice presents more risks than benefits [52,53]. Physical restraint can have serious physical consequences such as increased injury, morbidity, and mortality, as well as increased impairment of memory, language, loss of function, increased risk of incontinence, ulcers, and cognitive impairment [54]. A restraint-free nursing care environment has been recommended as the standard of care [56]. Accordingly, many interventions have been made in recent years to reduce the use of physical restraints in older people with dementia [57]. In Spain, following a direct observation study of physical restraint in long term care centers, rates are much higher (84.9%) than in other countries such as Northern Ireland, Canada, and Taiwan, with values between 62 and 68%, and the lowest in Germany, with 26.2% [58]. This lack of knowledge about patient safety and the use of physical restraints by healthcare professionals has been reported by many authors, not only in long-term care but also in hospitals and homes. Additional education is recommended for professionals working with people with dementia [58,59,60,61]. 

Regarding pain management, a survey on the knowledge and management of pain in older people with dementia among healthcare professionals showed that 58% of Spanish professionals feel that they have little knowledge of pain management in older people with dementia and only 14% have received specific training. Moreover, confidence in pain management decreased as the disease progressed to advanced stages, as 60% did not know which instruments were valid for pain assessment and whether there were any guidelines or standards of care for elderly people with advanced dementia [62]. Older people with advanced dementia also have pain, which is greater in the early stages than in the more advanced ones. The ability to express pain decreases in patients with dementia as the disease progresses due to the loss of cognitive and language skills. Despite this, however, the facial expression of pain in people with dementia is very homogeneous in both presence and intensity, thus allowing us to detect it using validated scales [63,64,65]. This may be partly attributed to knowledge deficits and negative attitudes of health care staff and informal caregivers towards pain, its assessment, and management in dementia [66]. Healthcare professionals should gain confidence and be able to distinguish signs and symptoms of pain from dementia-related behavioral changes. Interdisciplinary team assessment and communication are essential for proper pain management in the older with dementia [67].

The concept “Anticipation of needs” not only includes the management described above but also the individualization of care to anticipate healthcare decisions in the event that the patient loses decision-making capacity, either temporarily or permanently. These problems would be solved if elderly people with dementia had Advance Care Planning (ACP) [68]. 

Regarding the item “Persons with advanced dementia can fatigue or tire easily, and as a result, they usually need to lie down frequently”, it is well known that alterations in sleep patterns are common complaints in the elderly, especially those diagnosed with advanced dementia [69]. These patients experience difficulties falling asleep in 69% and sleeping excessively during the day in 76.8% [70]. In extreme cases, a complete reversal of the day/night sleep pattern can be observed with the main sleep period that occurs during the day [71]. This situation favors health personnel who associate the concept of fatigue directly with the quality of sleep [72]. 

In summary, the internal consistency, reliability, and difficulty indexes of the Spanish version of qPAD are similar to those obtained by versions obtained in other languages. However, a reformulation of the items with the lowest content validity or discrimination indexes and those showing difficulty with comprehension needs further studies in order to improve the qPAD for Spanish healthcare professionals prior to its use in clinical settings. 

## Figures and Tables

**Table 1:** Knowledge of palliative care in advanced dementia.

	CVI	Kappa	% Right Answers	% Wrong Answers	% “Don’t Know” Asnwers	Difficulty (Mean)
1. The best way to prevent weight loss for persons with advanced dementia is to keep them on their special medical diets (e.g., low fat, cardiac, renal).	0.92	0.92	50.97	35.92	13.11	0.51
2. It is possible to prevent pressure ulcers in persons with advanced dementia.	0.92	0.92	95.14	2.43	2.43	0.95
3. It is possible to prevent weight loss in most persons with advanced dementia.	1	1	52.43	29.13	18.44	0.52
4. Since persons with advanced dementia are so impaired, it is not likely that they are depressed.	1	1	93.20	3.40	3.40	0.93
5. One benefit of advanced dementia is that people no longer have pain.	1	1	96.12	0.97	2.91	0.96
6. When a person is resistive to “hands-on” care, it is best to stop what you are doing and come back later to try to complete the task.	1	1	85.44	5.82	8.74	0.85
7. Persons with advanced dementia should take showers just as other persons do.	1	1	66.99	21.84	11.16	0.67
8. Persons with advanced dementia cannot verbally tell us when they are hungry or thirsty.	1	1	65.53	24.76	9.71	0.65
9. Persons with advanced dementia can reposition themselves easily in their chairs.	1	1	90.78	3.88	5.34	0.91
10. Although persons with advanced dementia are incontinent, it is still possible to toilet them.	0.92	0.92	75.73	12.62	11.65	0.77
11. The sounds of music, meal service and conversations during dining do not generally pose problems for people with advanced dementia.	0.92	0.92	84.47	8.74	6.79	0.84
12. Persons with advanced dementia typically die from some sort of infection, such as pneumonia or a urinary tract infection.	0.92	0.92	83.01	6.80	10.19	0.83
13. Physical restraints decrease the chance that a person with advanced dementia will fall.	1	1	44.17	40.78	15.05	0.44
14. When people “call out” over and over again, it is best to not worry about this behavior because this is a common occurrence for persons with advanced dementia.	1	1	92.23	2.43	5.34	0.92
15. Persons with advanced dementia will never experience boredom.	0.85	0.84	93.20	0.97	5.83	0.93
16. If persons with advanced dementia resist (e.g., hit, bite, kick etc.) a brief change, it may be due to invasion of privacy.	0.92	0.92	88.35	2.91	8.74	0.88
17. Persons with advanced dementia should get pain medications around-the-clock, when needed.	0.85	0.84	36.41	49.03	14.56	0.36
18. “Anticipation of need” refers to addressing the needs of persons with advanced dementia through a daily schedule established by the facility where they live.	0.85	0.84	43.20	50.48	6.31	0.43
19. If a person with advanced dementia is unable to sleep at night, a sleeping medication should be considered first.	0.92	0.92	78.64	11.17	10.19	0.79
20. When persons with advanced dementia spit out their food, it is because they are not hungry.	1	1	90.29	1.46	8.25	0.90
21. Persons with advanced dementia really can’t convey or relate to caregivers if they are hungry, have pain, or need to use the bathroom.	0.92	0.92	30.10	59.22	10.68	0.30
22. Persons with advanced dementia can fatigue or tire easily, and as a result, they usually need to lie down frequently.	0.92	0.92	38.35	36.41	25.24	0.38
23. When persons with advanced dementia rapidly become more confused or display changes in behavior, it is likely that their dementia is getting worse.	0.92	0.92	54.37	31.07	14.56	0.54

**Table 2:** Attitudes towards palliative care in advanced dementia.

	CVI	Kappa	Mean
1. I believe my work experience enables me to discuss advanced dementia care with families.	0.92	0.92	3.5
2. I believe my education enables me to discuss advanced dementia care with families.	1	1	3.48
3. I believe it is important that caregivers provide families with information about end-of-life decisions.	1	1	4.77
4. Families are given consistent information about the consequences of their end-of-life care decisions.	0.92	0.92	3.38
5. Families are regularly included in ongoing discussions regarding advanced dementia care needs for their loved ones.	1	1	3.63
6. I frequently talk with my teammates about how we can change and improve the care for persons with advanced dementia.	1	1	3.54
7. I am regularly included in the care-planning for persons with advanced dementia.	1	1	3.26
8. My supervisor and team regularly listen to me regarding suggestions for persons with advanced dementia.	1	1	3.32
9. On most days, I am satisfied with my job of caring for persons with advanced dementia.	1	1	3.36
10. My input and opinion are valued regarding the needs of persons with advanced dementia.	1	1	3.45
11. On most days, I feel I’m part of the care team.	1	1	3.79
12. I enjoy providing care for persons who have advanced dementia.	1	1	3.87

## Data Availability

Data will be available upon request and reasoned justification.

## Supplementary Materials

The following supporting information can be downloaded at: https://www.mdpi.com/article/10.3390/healthcare10040656/s1, Supplementary S1: PARTE 1. Cuestionario sobre conocimientos; PARTE 2. Cuestionario sobre actitudes.
