# Supplementary material for: Knowledge and Attitudes towards Palliative Care: Validation of the Spanish Version of Questionnaire on Palliative Care for Advanced Dementia
Source: Healthcare (Basel). 2022 Mar 31;10(4):656. doi: 10.3390/healthcare10040656 (PMC9029205; doi:10.3390/healthcare10040656)
Supplement: Supplementary file 1 [file healthcare-10-00656-s001.zip › healthcare-1581918-supplementary.pdf]

PARTE 1. Cuestionario sobre conocimientos

Debe evaluar cada uno de estos aspectos de 1 a 4 (siendo 1 la peor puntuación y 4 la mejor) y dar una puntuación global (también de 1 a 4) para cada uno de dichos ítems.

1. La mejor manera de prevenir la pérdida de peso de las personas con demencia avanzada es mantener sus dietas terapéuticas (por ejemplo, baja en grasa, cardiosaludable, renal).

2.Es posible prevenir las úlceras por presión en personas con demencia avanzada.

3. Es posible prevenir la pérdida de peso en la mayoría de las personas con demencia avanzada.

4.Como las personas con demencia avanzada presentan un gran deterioro cognitivo, no es probable que estén deprimidos.

5.Un aspecto positivo de la demencia avanzada es que las personas ya no tienen dolor.

6.Cuando una persona ofrece resistencia a cuidados que suponen un contacto manual, es mejor dejarlo en ese momento y retomarlo más tarde.

7.Las personas con demencia avanzada han de ser bañadas con una técnica similar a la utilizada con otras personas.

8.Las personas con demencia avanzada no pueden expresar verbalmente cuándo tienen hambre o sed.

9.Las personas con demencia avanzada pueden recolocarse por sí mismos fácilmente en las sillas.

10.Aunque las personas con demencia avanzada tengan incontinencia, aún se les puede ayudar a utilizar el retrete.

11. La música, el ajetreo en el reparto de las comidas y las conversaciones durante las comidas generalmente no plantean problemas para las personas con demencia avanzada.

12.Las personas con demencia avanzada suelen morir de algún tipo de infección, como las neumonías o las infecciones del tracto urinario.

13.El uso de sujeciones físicas disminuye el riesgo de caídas en personas con demencia avanzada.

14.Cuando las personas con demencia avanzada gritan de manera continua, no hay que preocuparse porque este comportamiento es común en este tipo de patología.

15.Las personas con demencia avanzada no se aburren nunca.

16.Si una persona con demencia avanzada se resiste (con golpes, mordiscos, patadas, etc.) al cambio de pañal, puede deberse a que lo percibe como una invasión de su intimidad.

17.Las personas con demencia avanzada deben recibir fármacos contra el dolor a demanda, cuando los necesiten.

18.Para anticiparse a las necesidades de cuidado de las personas con demencia avanzada es necesario que desde el centro residencial en el que viven se establezca un plan general de actividades diarias.

19. Si una persona con demencia avanzada no puede dormir por la noche, lo primero se debe contemplar es la administración de un fármaco para dormir.

20.Cuando las personas con demencia avanzada escupen la comida es porque no tienen hambre.

21.Las personas con demencia avanzada no pueden transmitir o explicar a sus cuidadores que tienen hambre, dolor, o necesitan ir al baño.

22.Las personas con demencia avanzada se cansan fácilmente, y por tanto necesitan que les acuesten para descansar a menudo.

23.Cuando las personas con demencia avanzada presentan confusión repentina o muestran cambios de comportamiento, es probable que su demencia está empeorando.

PARTE 2. Cuestionario sobre actitudes

Debe evaluar cada uno de estos aspectos de 1 a 4 (siendo 1 la peor puntuación y 4 la mejor) y dar una puntuación global (también de 1 a 4) para cada uno de dichos ítems.

1. Creo que mi experiencia laboral me capacita para hablar del cuidado de personas con demencia avanzada con sus familiares

2.Creo que mi formación me capacita para hablar del cuidado de personas con demencia avanzada con sus familiares

3.Creo que es importante que los cuidadores profesionales ofrezcan a los familiares información sobre decisiones al final de la vida.

4.Los familiares reciben información coherente y veraz sobre las consecuencias de sus decisiones sobre los cuidados al final de la vida.

5.Se tiene en cuenta a los familiares en el seguimiento de las necesidades de cuidados de sus seres queridos con demencia avanzada.

6.Con frecuencia hablo con mis compañeros de equipo sobre cómo podemos cambiar y mejorar el cuidado de las personas con demencia avanzada.

7.Me incluyen habitualmente en la planificación de la atención a personas con demencia avanzada.

8.Mi supervisor/a y mi equipo escuchan regularmente mis sugerencias sobre el cuidado de las personas con demencia avanzada.

9.La mayoría de los días estoy satisfecho con mi trabajo en el cuidado de las personas con demencia avanzada.

10.Se valora mi aportación y mi opinión sobre las necesidades de las personas con demencia avanzada

11.La mayoría de los días me siento parte del equipo de cuidados.

12.Disfruto cuidando a las personas que tienen demencia avanzada.
